# Retraction: CTL–doxorubicin (DOX)–gold complex nanoparticles (DOX–AuGCs): from synthesis to enhancement of therapeutic effect on liver cancer model

**DOI:** 10.1039/d5na90068a

**Published:** 2025-09-29

**Authors:** Qiqian Liu, Hui Liu, Pasquale Sacco, Nadia Djaker, Marc Lamy de la Chapelle, Eleonora Marsich, Xiaowu Li, Jolanda Spadavecchia

**Affiliations:** a CNRS, UMR 7244, NBD-CSPBAT, Laboratoire de Chimie, Structures et Propriétés de Biomatériaux et d'Agents Thérapeutiques Université Paris 13 Sorbonne Paris Nord Bobigny France jolanda.spadavecchia@univ-paris13.fr; b Department of Hepato-biliary Surgery, Shenzhen University General Hospital, Guangdong Provincial Key Laboratory of Regional Immunity and Diseases, Carson International Cancer Shenzhen 518055 China; c Department of Life Sciences, University of Trieste Via L. Giorgieri 5 I-34127 Trieste Italy; d Department of Medicine, Surgery and Health Sciences, University of Trieste Piazzale Europa 1 I-34127 Trieste Italy; e IMMM – UMR 6283 CNRS, Université du Mans Avenue Olivier Messiaen 72085 Le Mans Cedex 9 France

## Abstract

Retraction of “CTL–doxorubicin (DOX)–gold complex nanoparticles (DOX–AuGCs): from synthesis to enhancement of therapeutic effect on liver cancer model” by Qiqian Liu *et al.*, *Nanoscale Adv.*, 2020, **2**, 5231–5241, https://doi.org/10.1039/D0NA00758G.

The Royal Society of Chemistry, with the agreement of the named authors, hereby wholly retracts this *Nanoscale Advances* article due to concerns with the reliability of the data.

Following the previous publication of a correction to correct errors in Fig. 1, the Royal Society of Chemistry has been notified about the conclusion of an institutional investigation carried out by the CNRS and Sorbonne Paris Nord University recommending retraction of this *Nanoscale Advances* article.

The validity of the new data published in the correction is disputed as the corrected histogram cannot be representative of the particles represented in the corrected TEM image. In addition, the nanoparticle release curves as a function of time presented in Fig. S2 are falsified, as they are duplicates of at least three previous publications corresponding to different nanoparticles.

In view of the falsification of data, the institution recommends that this publication, which has already been corrected, be retracted.

This retraction supersedes the information provided in the correction related to this article and the correction is no longer valid.

The contribution of the authors Pasquale Sacco and Eleonora Marsich, affiliated with the University of Trieste, did not concern the acquisition, analysis and interpretation of the disputed experimental data, but it concerned exclusively the physical–chemical characterization of the CTL polysaccharide, more specifically the determination of its intrinsic viscosity and the degree of substitution, which was used in this study for the preliminary synthesis of nanoparticles.

The authors were informed about the retraction of the article. Jolanda Spadavecchia, Qiqian Liu, Nadia Djaker, Hui Liu, Xiaowu Li and Marc Lamy de la Chapelle have not agreed with the decision, Pasquale Sacco and Eleonora Marsich have agreed to the retraction.

Signed: Pasquale Sacco and Eleonora Marsich

Date: 22nd September 2025

Retraction endorsed by Jeremy Allen, Executive Editor, *Nanoscale Advances*

